# The Role of Growth Hormone on Ovarian Functioning and Ovarian Angiogenesis

**DOI:** 10.3389/fendo.2019.00450

**Published:** 2019-07-16

**Authors:** Jesús Devesa, Diego Caicedo

**Affiliations:** ^1^Scientific Direction, Medical Center Foltra, Foundation Foltra, Teo, Spain; ^2^Department of Vascular Surgery, Health Research Institute of Santiago de Compostela (IDIS), University Hospital of Santiago de Compostela, Santiago de Compostela, Spain

**Keywords:** growth hormone, IGF-1, leptin, kisspeptin, GnRH, puberty, menopause, ovarian angiogenesis

## Abstract

Although not yet well-understood, today it is clear that Growth Hormone (GH) exerts a relevant role in the regulation of ovulation and fertility; in fact, fertility is lower in women with GH deficiency (GHD), and GH receptors (GHR) and GH mRNA have been found in the ovary since the onset of follicular development in humans. However, despite the strong evidence of GH in the regulation of fertility, many aspects of GH actions at this level are still not well-established, and it is likely that some controversial data depend on the species analyzed, the dose of the hormone and the duration of use of GH. Folliculogenesis, ovulation, and corpus luteum formation and maintenance are processes that are critically dependent on angiogenesis. In the ovary, new blood vessel formation facilitates oxygen, nutrients, and hormone substrate delivery, and also secures transfer of different hormones to targeted cells. Some growth factors and hormones overlap their actions in order to control the angiogenic process for fertility. However, we still know very little about the factors that play a critical role in the vascular changes that occur during folliculogenesis or luteal regression. To promote and maintain the production of VEGF-A in granulosa cells, the effects of local factors such as IGF-I and steroids are needed; that VEGF-A-inducing effect cannot be induced by luteinizing hormone (LH) or chorionic gonadotropin (CG) alone. As a result of the influences that GH exerts on the hypothalamic-pituitary-gonadal axis, facilitating the release of gonadotropins, and given the relationship between GH and local ovarian factors such as VEGF-A, FGF-2, IGF-1, or production of sex steroids, we assume that GH has to be a necessary factor in ovarian angiogenesis, as it happens in other vascular beds. In this review we will discuss the actions of GH in the ovary, most of them likely due to the local production of the hormone and its mediators.

## Introduction

**Figure 1:**
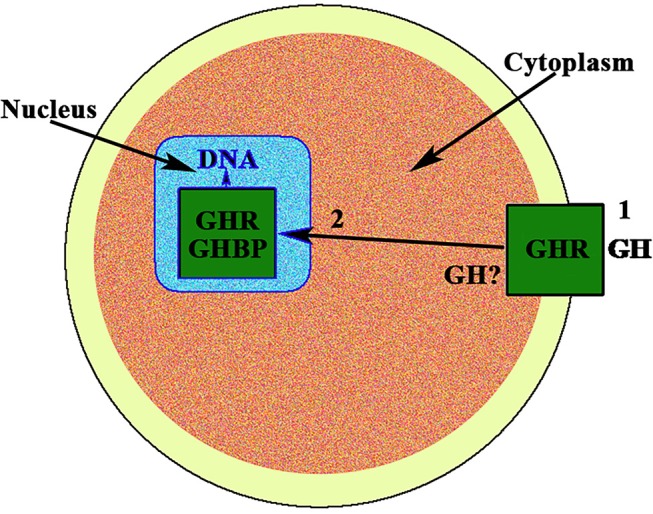
GH receptor in the nucleus of the oocyte. In the nucleus of the oocyte, the GH receptor (GHR) and the carrier protein GHBP have been found. This means that GH, after interacting with its membrane receptor (1), has allowed the internalization of both, GH and GHR, (2) and then the receptor is translocated to the cell nucleus where it would act as a gene transcription factor. The possibility exists that the own GH is expressed in the oocyte.

Classically, since its discovery, isolation and administration to a pituitary dwarf (1), the pituitary growth hormone (GH) has been considered a metabolic hormone that, in addition, has specific effects on growth until puberty ends. This concept remains widely valid, despite the many and very important physiological roles that GH plays in the human body, now well-known, in virtually all tissues and organs (2). A schematic representation of these multiple functions of GH can be seen in Caicedo et al. (3), Figure 1 in this reference. Moreover, for years, we have known that apart from the endocrine GH expressed and released from the pituitary gland, there are expressions of the hormone in many cells and tissues, where it plays autocrine/paracrine roles (4), the meaning of which is still little understood in many cases, although its importance is beyond doubt in situations such as for example, the induction of cell proliferation or survival, or inducing the expression of glucose transporters which allow the uptake of glucose needed for the production of energy by cells. In addition, after interacting with its membrane receptor, extracellular GH is translocated to the nucleus (5), where the hormone interacts with its receptor (GHR), and its binding protein GHBP (the extracellular domain of GHR), inducing changes in transcriptional activity (5, 6). These findings contradicted the classical concept about the fact that protein hormones only acted on their receptors in the cell membrane. More recent studies introduce more complexity about the mechanisms of action of GH at the cellular level. It has been demonstrated, in pigs, that GH administration induces the translocation of GHR to cell nuclei *in vivo*, which may indicate that the nuclear GHR exerts functions that we do not yet know (7), although presumably, it acts there as a transcriptional factor.

In any case, the supply of GH to cells or tissues needs an adequate blood flow, and the hormone plays a very important role in the formation of new blood vessels or the recovery of damaged vasculature (3).

In the case of gonadal function in females, it seems to be of interest that, to our knowledge, the first description of the existence of a strong immunoreactivity for the GH receptor at the nuclear level, was described in 1990 in rat oocytes (8) (Figure 1). The same study found a very large distribution of the GHR/GHBP in practically all the reproductive system of the rats analyzed, suggesting that GH could play important and direct actions on reproduction, but also in the normality and integrity of the endometrial vascular system (8). Interestingly, these authors also found that this GHR/GHBP immunoreactivity was clearly higher in the ovarian granulosa cells from 10 days old rats than in granulosa cells from adult animals. This could be related to the fact that the pituitary secretion of GH gradually decreases along aging (9), but also with the fact that vasculature suffers progressive damage along life (3), with the subsequent decrease of blood supply to the ovary.

In this review, we will analyze the role played by GH for a normal ovarian function, and also the role of ovarian angiogenesis in ovarian functioning, as well as the effects of aging on both processes.

## Ovarian Functioning

### Ovarian Cycle

The ovarian gland suffers continuous physiological and morphological changes from embryogenesis until menopause and during the physiological ovarian cycle, most of them clearly related with the intervention of GH, apart from pituitary gonadotropins. As is logical, these changes must be accompanied by changes in the blood supply to ensure adequate supply, not only of nutrients but also of hormones and factors involved in these changes. This, in turn, means that the number, size, distribution, and functionality (permeability) of the blood vessels must also change considerably throughout the life of this gland, not only at the beginning of puberty but also daily, in each menstrual cycle, and during the reproductive life of a woman, until menopause. Therefore, ovarian angiogenesis is also a critically regulated process, both for ovulation and for development and function of the corpus luteum (CL).

In fact, once the puberty begins, folliculogenesis, ovulation, and CL formation and maintenance are processes that are critically dependent on angiogenesis. Irrigation of the ovary comes from a direct branch of the abdominal aorta, the ovarian artery, although this artery is anastomosed with a second source of ovarian irrigation, the ovarian branches of the uterine arteries, coming from the internal iliac arteries. Although these main sources of blood supply to the ovary do not change physiologically, an intricate network of pre-existing, initially non-functional, arterioles and newly formed capillaries arise from them, and continually change to support the continuous changes that occur in follicular development, ovulation, luteogenesis, and luteolysis. This is in a clear contrast with that occurring in other organs and tissues, in whom, physiologically, the vascular system does not suffer significant changes (10).

**Figure 2:**
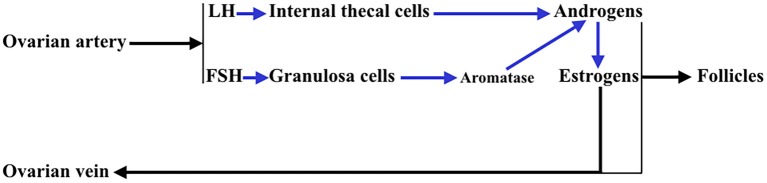
Interactions between the theca cells of the internal layer and the granulosa cells in a growing follicle. Under the stimulus of pituitary LH, the theca cells produce androgens that reach the granulosa cells and in these, under the control of pituitary FSH, that activates aromatase, they are aromatized to estrogens (mainly estradiol). These estrogens are released into the systemic circulation and follicular fluid. Likewise, a small part of the androgens produced in the thecal cells pass into the systemic circulation.

In addition, the ovary is an endocrine gland from which sex steroids are released into the blood under the stimulation of pituitary gonadotropins (Gns), to perform very important functions in the female body, but also to regulate in the ovary the sequence of processes that lead to ovulation and luteogenesis. This concept is schematically represented in Figure 2.

### Growth Hormone and Ovarian Functioning

As described in the Introduction, the presence of GHR in rat oocytes, and also in practically the entire reproductive system of these animals was found almost 30 years ago (8). This was the first indication that GH played a very important role in the reproductive system of this species, something posteriorly verified in other animal models (11). Later studies showed that there is a genetic expression of GHR in cumulus cells, mature oocytes and preimplantation human embryos, in which there is a high expression of GHR from the 4-day morula onwards (12). This study led to the conclusion that, in humans, GH plays a role in the maturation of the oocyte and embryogenesis, from its early stages. GH and GHR have been found in human ovaries from fetuses and adults (13), where they play a very important autocrine/paracrine role.

**Figure 3:**
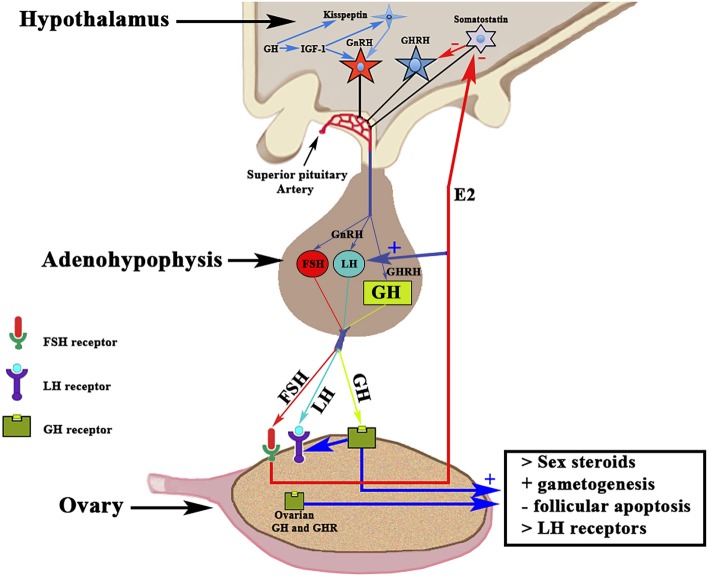
Schematic representation of the Hypothalamic-Pituitary-Ovarian axis and the effects of GH on ovarian functioning. The activation of the GnRH pulse generator leads to the release of GnRH in the portal blood from which it reaches the gonadotropic cells in which it induces the release of FSH and LH (in proportions and amounts variable throughout a menstrual cycle) in the systemic circulation. Both gonadotropins interact with their ovarian receptors, triggering the previously described effects. The release of pituitary GH is induced by hypothalamic GHRH, which is negatively regulated by somatostatin. Systemic GH interacts with its ovarian receptor GHR and induces the positive regulation of LH receptors. The activation of GHR induces, throughout its effects on thecal cells, an increase in the production of sex steroids, mainly estradiol (E2), which in addition to its actions at the ovarian level, is released into the general circulation. Estradiol increases the pituitary release of LH, and also acts by inhibiting the hypothalamic release of somatostatin, which allows GHRH to be released into portal blood and stimulate pituitary synthesis and release of GH. GH and its GHR also are produced in the ovary; therefore, the ovarian actions of GH may also depend on the GH–GHR interaction. GH acts on the oocyte and the survival of the ovarian follicles. GH is also produced in the brain, where it stimulates the synthesis of IGF-1 that activates the GnRH pulse generator. In addition, both brain GH and IGF-1 stimulate kisspeptin neurons to release kisspeptin, a key factor in the activation of the GnRH pulse generator. +, stimulation; –, inhibition.

Although most of the GH effects on the ovarian function are exerted by the hormone locally produced in the ovaries, systemic GH released by the pituitary gland or exogenously administered, also plays an important role in the normal function of the female gonad and reproduction, as previously reviewed by Hull and Harvey (14). Gonadal steroids participate in the hypothalamic regulation of pituitary GH secretion (9); GH pulse amplitude increases in hypogonadal prepubertal girls with Turner's syndrome when they receive E2 (15). In turn, GH participates in the regulation of puberty and fertility through changes in Gns secretion, directly or via IGF-1 (16, 17). Hypothalamic neurons producing the gonadotropin-releasing hormone (GnRH) express IGF-1 receptors, therefore Gns secretion may be regulated, at least partially, by the GH/IGF-1 axis (16, 18, 19). IGF-1 has been shown to stimulate GnRH promoter activity *in vitro* (20, 21). IGF-1 has been found in the brain, and there its expression is stimulated by GH, at least in human neural stem cells (22). IGF-1 is able, in rats, to stimulate the biosynthetic activity of pituitary gonadotrophs *in vitro* (18), and it has been shown that this peptide acts directly on GnRH neurons for the regulation of puberty in rats (19), although it can also act on kisspeptin neurons, which play a key role in regulating the activity of GnRH neurons [for review, see (19)]. A very recent study reveals that GH directly exerts effects on kisspeptin neurons located in the anteroventral periventricular and rostral periventricular nuclei via the signal transducer and activator of transcription 5 (STAT5), while the hormone lacks any effect on kisspeptin neurons located at the arcuate nucleus (23). These data agree with clinical data indicating that an appropriate secretion of GH is needed for sexual maturation and maintenance of reproductive functions, while GH deficiency may affect the beginning of the puberty and can produce infertility. In humans, the interaction GH–GHR in the ovary promotes the synthesis of sex steroids and induces gametogenesis, inhibits follicular apoptosis, and upregulates ovarian receptors for LH (14, 24, 25). These concepts are schematized in Figure 3. GH replacement therapy restores normal ovarian function in women with GH deficiency (26), in which the onset of puberty is delayed and reproductive function altered, and in infertile eugonadal women with GH deficiency in whom GH treatment restores fertility with successful pregnancies (27).

### Growth Hormone and Puberty in Females

**Figure 4:**
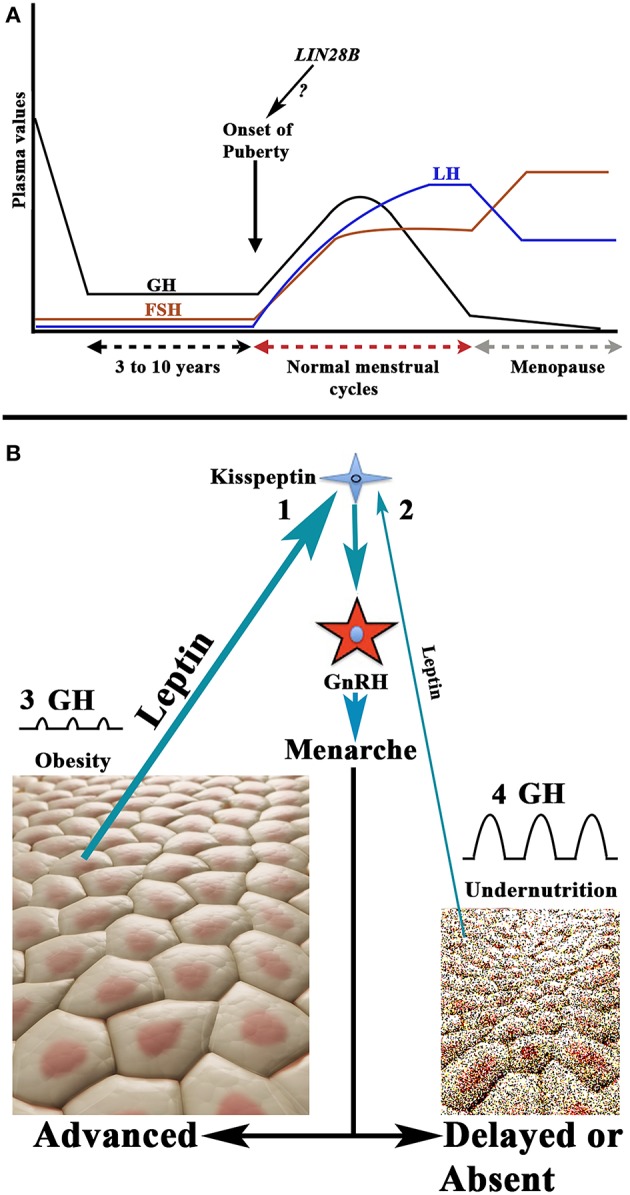
Schematic representation of the secretion of gonadotropins and GH from childhood to aging, and the relationship between nutritional status and the onset of puberty. **(A)** Plasma GH, LH, and FSH values from childhood to aging in a normal woman. Note that during childhood the FSH levels, although low, are permanently higher than plasma levels of LH. The onset of puberty is characterized by a nocturnal increase of LH on the plasma values of FSH; then LH is always at higher values than that of FSH (with the exception of the first days of the follicular phase and the last days of the luteal phase during a menstrual cycle). At menopause, the secretion of both gonadotropins increases considerably, and the release of FSH is markedly higher than that of LH. The evolution of the secretion of GH follows a pattern similar to that of LH up to 18–20 years. From this age it decreases gradually until it is practically undetectable in old age. Curiously, the highest secretion of GH occurs when puberty begins. As shown in the figure the possibility exists that the *LIN28* gene activation may be responsible for the onset of puberty. **(B)** There is a clear relationship between the body fat mass and the activation of the GnRH pulse generator. Adipocytes release leptin which, among other actions, stimulates the production of kisspeptin who, in turn, activates the production and secretion of GnRH. This leads to menarche. Therefore, the greater amount of body fat, the higher secretion of leptin (1) and the lower age of onset of the puberty, while undernutrition leads to decreased leptin production (2) and this leads to delayed or absent puberty onset. In these opposite situations, the rate of secretion of GH does not take parallel to the onset of puberty. In obesity, the secretion of GH is considerably reduced or absent (3), whereas in girls with low fat mass the secretion of GH is markedly increased (4). This is probably due to the fact that the increase in the fat mass produces an increase in the hepatic production of IGF-1, which induces the hypothalamic release of somatostatin, but also directly inhibits the pituitary release of GH, while undernutrition prevents the synthesis of IGF-1 in the liver, therefore leading to an increase in GH secretion.

For years, it has been known that the onset of puberty is characterized by a change in the secretion pattern of pituitary gonadotropins. During childhood both gonadotropins circulate in low levels, and FSH secretion clearly predominates over LH secretion, whereas when puberty begins, not only do the plasma levels of both gonadotropins increase, but this pattern is also inverted. First, only during the night is the secretion of LH higher than that of FSH, and then throughout the day the menarche appears and menstrual cycles begin (28, 29). These pubertal changes occur as a consequence of the activation of the GnRH pulse generator, previously practically quiescent, produced by neurotransmitters acting on GnRH neurons (29) and the stimulatory effect of hypothalamic kisspeptin on them (30). The question is: why does this activation of the GnRH pulse generator usually occur at a certain age, highly variable among girls, during development? It is well-known that the onset of the puberty shows great changes among different ethnic populations throughout the world, which indicates that there is a genetic control on the timing of puberty (31). Although most likely the onset of puberty is polygenic, in a high number of girls a study identified the Single Nucleotide Polymorphism (SNP) rs314276 in the intron 2 of *LIN28B* gene, located on chromosome 6, as the first genetic marker associated with menarche (32), although other genes also contribute to the physiology of this important event in the development (33, 34). Figure 4A shows the changes in gonadotropins and GH secretion from childhood to old age.

Besides its genetic determinant, the onset of the puberty is conditioned by the nutritional status of the individual, something logical as a minimum availability of available energy is necessary to face the beginning of a reproductive stage. In fact, increased fat mass has been associated with precocious puberty (35, 36), while undernutrition or weight loss lead to delayed menarche or amenorrhea produced by hypogonadotropic hypogonadism (37). In line with this, leptin, a hormone produced by the adipose tissue (38), to regulate body fat mass by inhibiting food intake by stimulating the satiety center, is related to the onset of puberty. The reason is that increased fat mass would lead to increased leptin production, and since obesity induces precocious puberty, leptin has been considered as a metabolic signal to the reproductive system (39). More recent studies indicate that high levels of leptin in prepubertal girls are clearly associated with menarche at younger ages (40). The permissive role of leptin in the onset of puberty has been suggested to be mediated by kisspeptin neurons, given that alterations of the *KISS1* gene and the kisspeptin receptor have been associated with leptin-deficient production (41). Moreover, reproductive alterations can be recovered by leptin administration, both in humans and animals. Therefore, there seems to be a leptin-kisspeptin-GnRH pathway that carries metabolic information to the centers that regulate reproductive function (42). These concepts are shown in Figure 4B.

Once we have briefly analyzed how puberty starts in girls, it is appropriate to assess whether GH plays a role in this process. The pituitary secretion of GH increases sharply as puberty approaches; consequently, the rate of growth velocity also increases to more than double or triple the values observed throughout childhood (2). This apparent relationship between increased GH secretion and the onset of puberty led to the assumption that GH could act as a co-gonadotropin that increases the effects of FSH and LH on the production of ovarian sex steroids (43). In this line, GH-deficiency has been identified as the only cause of primary amenorrhea in three adolescent women in whom the secretion of gonadotropins was normal, suggesting that GH would play a complementary role to gonadotropins for the onset of menarche (44). As stated above, GH-deficiency negatively affects ovarian function in humans delaying sexual maturation (26) and fertility (27), a situation that is reversed with GH replacement therapy.

It has been postulated that the increase of GH secretion when puberty begins depends on the activation of the GnRH pulse generator (43); the consequent increase in the secretion of LH would stimulate the production and secretion of ovarian sex steroids, which, in turn, increase noradrenergic pathways that acting on alpha-2 receptors block somatostatin secretion and allow the hypothalamic release of GHRH (9); this would lead to an increase in the synthesis and secretion of pituitary GH and would explain the increase in plasma levels of this hormone and the rate of growth during puberty (Figure 4A). Although this possibility is real, the fact that GH and IGF-1 stimulate, in rats, the activity of the GnRH promoter *in vitro* (18, 19) and kisspeptin neurons (23), may indicate that the activation of the GnRH pulse generator is an event that follows the increased secretion of GH/IGF-1 that occurs when pubertal maturation begins. Another possibility is that both processes occur in parallel and, physiologically, each one of them provides positive feedback to the other. It would be of interest to investigate whether GH might participate in the regulation of the expression of *LIN28B* gene or other genes involved in the onset of puberty.

As described above, the onset of puberty in girls is also dependent on the nutritional status. A recent meta-analysis indicates that obesity leads to early onset of puberty (45). This corroborates other studies that relate the increase in fat mass and early menarche (35, 36, 46–50). However, obesity leads to a reduction or absence of GH secretion (2, 37, 50) (Figure 4B), suggesting that GH does not play a role in the onset of puberty. Despite the lack of a significant secretion of GH, although a residual secretion of the hormone in this situation cannot be discarded, the growth rate is normal or even higher in obese girls than in girls with normal fat mass (2). This can be explained by the higher levels of IGF-1 in obesity, which is the real hormone responsible for growth (2), just as early puberty in these cases can be explained by the increase in IGF-1 itself (17) and the high levels of leptin produced by the excessive fat tissue (40) (Figure 4B).

In summary, the onset of puberty in girls is a very complex process in which many factors participate. Among them, genetic factors, nutritional status, environmental factors, ethnicity, but also hormonal factors such as GH/IGF-I and leptin. GH acts in conjunction with gonadotropins and perhaps is an inducer of the activation of the GnRH pulse generator.

### Growth Hormone and Menstrual Cycles

GH is a hormone that plays a very important role in the course of the processes that during a normal menstrual cycle culminate in the maturation and release of the oocytes; this function can be performed by the systemic hormone or the one produced in the ovary itself, but it can also depend on the ovarian IGF-1 induced by GH, or independently produced in this gland. The attribution of a specific effect to one of these hormones or to the GH/IGF-1 system is generally difficult to be established, as most of the data on the effects of GH and IGF-1 at the ovarian level come from cultured cells or studies in animals, and the effects seem to be different depending on the species. For example, it has recently been shown in ovarian sheep cultures, that IGF-1 is present in all stages of follicular development (51). In this study, IGF-1 was found in oocytes and GCs of antral follicles, acting synergistically with FSH to stimulate oocyte growth, increasing the number of fully developed oocytes and increasing the immunoreactivity of the LH receptor in GCs (51). This effect of IGF-1 seems to be dependent on GH, at least in bovines (52). Data from studies in cultured human GCs indicate that GH stimulates the production of IGF-2, which suggest that IGF-2 may be also a mediator of GH effects in follicles acting through the receptor for IGF-1 (53).

In any case, data from studies in women indicate that the GH/IGF-1 system plays a very important role during the human menstrual cycle.

To produce a viable normal embryo, a sequence of perfectly linked processes must occur in the post-menarchal ovary. These are: steroidogenesis, folliculogenesis, oocyte maturation, and luteogenesis. After menarche, oocytes secrete a number of factors that act on the surrounding GCs to regulate the development and function of these cells (54, 55); this allows the primary follicles to develop into secondary follicles and then grow to the preantral and antral stages, under the control of pituitary Gns, but also GH, as *in vivo* studies demonstrated that this hormone seems to be necessary for achieving an optimal maturation and survival of developing follicles (14, 56, 57), and increases ovarian weight in some species. In contrast, the absence of effects of GH, as occurs in knockout mice for GHR, leads to these animals to have few primary, secondary, preantral and antral follicles, and increased follicular atresia (25, 58).

**Figure 5:**
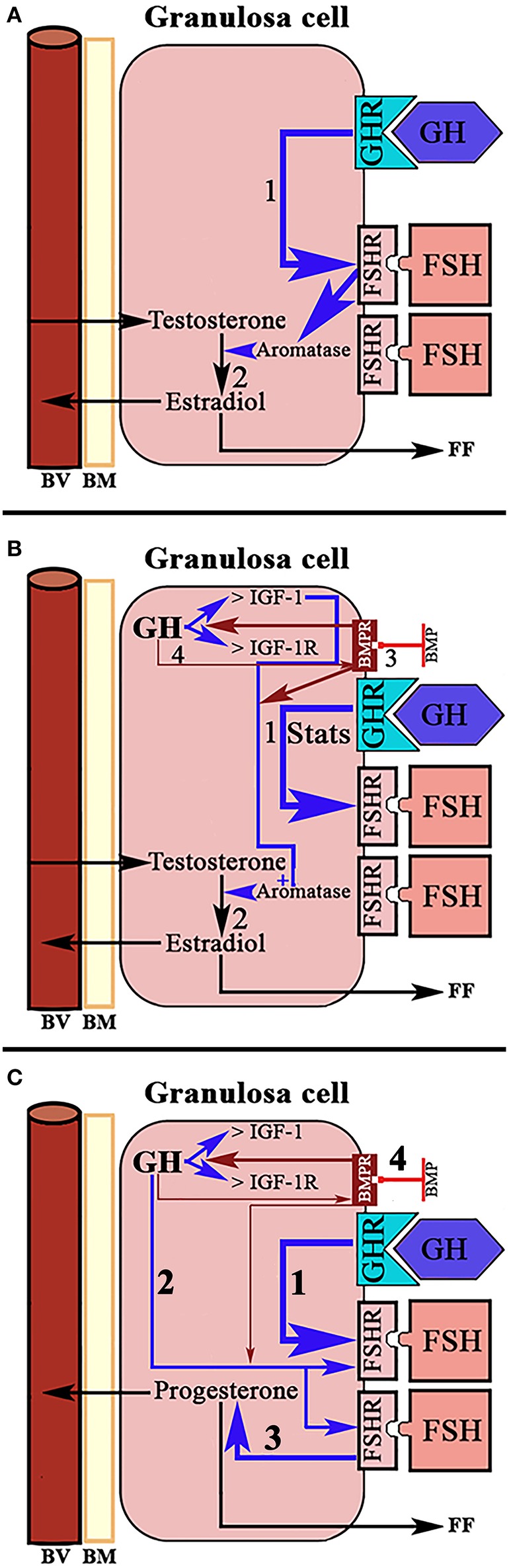
Schematic representation of the actions of GH/IGF-1 in the ovarian granulosa cells. **(A)** In normal women, administration of GH in the early follicular phase exerts a synergistic effect with FSH on the production of estradiol. GH up-regulates the receptors for FSH (1) and increases the activation of aromatase by this hormone. The result is increased formation of estradiol from testosterone from the theca cells (2). **(B)** In rats, it has been shown that the up-regulation of FSH receptors induced by GH is mediated through activation of Signal Transducers and Activators of Transcription STATS (1). Ovarian GH increases the expression of IGF-1 and its receptor, and is IGF-1 which increases FSH-dependent aromatase activity (2). Bone morphogenetic proteins (BMP) suppress the expression of the ovarian GH/IGF-1 axis (3), therefore inhibiting the aromatization of testosterone to estradiol. In turn, GH/IGF-1 down-regulate the expression of BMP receptors (4). Blue arrows: stimulation. Red arrows, inhibition. **(C)** Systemic GH (1) and/or ovarian GH (2) increase progesterone production in ovarian granulosa cells induced by FSH (3), while BMP blocks this effect of GH (4). Blue arrows, stimulation; Red arrows, inhibition; BV, blood vessel; BM, basement membrane; FF, Follicular fluid.

A normal menstrual cycle begins with a predominant secretion of FSH over LH. In this situation, growing preantral follicles TCs, stimulated by LH, produce androgens, mainly androstenedione, which reach GCs where they are converted in E2 by the action of an FSH-dependent aromatase (Figure 2). Years ago it was found that in 24 normal patients, with normo-ovulatory cycles, short-term GH administration exerted a synergistic effect on the FSH-induced follicular steroidogenesis, in terms of increased E2 production during the early follicular phase, significantly higher than that induced by FSH injection without GH administration, while GH alone was unable to induce any steroidogenic response, as happened when saline was given to these women (59). This GH effect might be attributed to an action of the hormone on the activity or expression of aromatase (Figure 5A). However, it has been demonstrated that GH inhibits FSH-induced aromatase activity via an IGF-1 independent pathway, therefore inhibiting E2 synthesis in rat GCs, while, as demonstrated many years ago, IGF-1 stimulates aromatase activity (60). It has been shown that GH increases IGF-1 and its receptor, and receptors for FSH in rat GCs via phosphorylation of Signal Transducers and Activators of Transcription (STATS), while the family of bone morphogenetic proteins (BMP) inhibits IGF-1 effects on FSH-induced E2 production by suppressing the expression of the ovarian GH/IGF-1 (61) (Figure 5B); curiously, the GH/IGF-1 system down-regulates the expression of BMP receptors, and GH upregulates inhibitors of BMP signaling, therefore negatively affecting BMP signaling pathways (61). Regarding P4 synthesis in GCs, it is induced by FSH and GH enhances this effect through induction of steroidogenic acute regulatory protein (StAR), cytochrome P450 and 3ß-hydroxysteroid dehydrogenase, but this is blocked by BMP (Figure 5C). Therefore, and since the expression of BMP members changes throughout a menstrual cycle, it seems that the relationship between GH/IGF-1 and BMP signal intensities, and that the type of them, plays a key role in the regulation of Gns-dependent steroidogenesis in follicles. Moreover, BMP also participate in the regulation of the hypothalamic secretion of GnRH and ovarian sensitivity to Gns (62). These effects of BMP, particularly BMP-15, have been also seen in humans (63, 64).

In humans, as in many other species, GH seems to play a direct role in the nuclear maturation of oocytes (12, 65). In human oocytes, the receptor for GH has been detected in the membrane, in cumulus cells (66) and in the nucleus in mature ovaries (13), a fact that confirms that GH has to act at this level improving nuclear maturation and the expansion of cumulus cells, as has been demonstrated in primates (67), and also improving the cytoplasmic maturation of mature oocytes (68).

**Figure 6:**
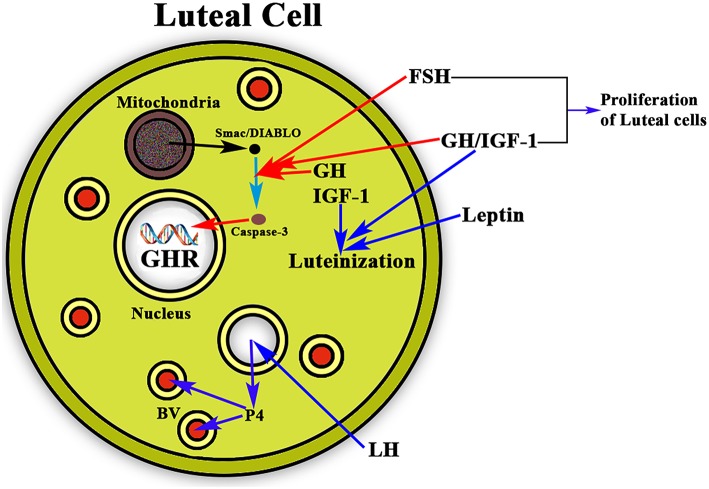
Schematic representation of the actions of GH/IGF-1 in the ovarian corpus luteum. The figure shows a luteal cell in which the GHR can be see inside the nucleus. The production of progesterone (P4) is induced by LH, then P4 enters the bloodstream (BV; blue arrows) for playing very important functions in the body, mainly if conception exists, but also acting at many other levels, even in the brain. As shown in the figure, GH and IGF-1 are expressed in the luteal cells where they act synergistically with leptin for inducing the luteinization of the follicle that has ovulated (blue arrows). In addition, GH/IGF-1 together with FSH inhibit the possible apoptosis of the luteal cells (red arrows) by inhibiting the proapoptotic activity of caspase-3, which is stimulated by Smac/DIABLO released from the mitochondria. Moreover, GH/IGF-1 together with FSH induce the proliferation of luteal cells.

Once ovulation occurred there is the need of maintaining the corpus luteum and the secretion of the needed amount of P4 from it if pregnancy occurs, until the placenta begins to produce its own P4. This allows implantation and avoids abortion. It has been shown that leptin acts synergistically with GH and IGF-1 in the luteinization of GCs, which begins before follicular disruption and ovulation (69). In this context, GH also seems to play a very important role as proliferative and antiapoptotic factor. GH together with FSH stimulates the proliferation of luteinized GCs (70) and avoids the apoptosis of these cells (Figure 6). The detection of the GHR in human luteal cells (8) supports the postulate that GH exerts a role in the important functions of the corpus luteum.

In addition to the important actions that the GH/IGF-1 system exerts on the ovaries, GH also facilitates the development of the most appropriate maternal environment, acting in the uterus very early in gestation. Both GH and its receptor are expressed in the uterus of pregnant and non-pregnant women [for review, see (2)]. GH induces uterine growth, thus facilitating implantation. In fact, pregnancy maintenance requires significant uterine hypertrophy. Clinical studies carried out in women with thin endometrium indicate that patients treated with GH developed greater endometrium thickness on day 3, higher implantation rates and greater clinical pregnancy rates than the untreated control patients (71). These effects have been attributed to the fact that the hormone, via JAK2/STAT5 pathway, promotes the proliferation of endometrial cells and increases uterine vascularization (through the regulation of VEGF-A expression); these GH actions could be mediated by the autocrine IGF-1 or the systemic IGF-1. As it has been described above GH facilitates implantation, most likely by increasing the production of matrix metalloproteinases and stimulating trophoblast cell proliferation which allows blastocyst cavity formation and invasion of the endometrium (72).

In summary, the GH/IGF-1 system (endocrine and autocrine or paracrine, or all of them) plays a very important function in practically all the physiological events occurring during a normal menstrual cycle, even facilitating the implantation and development of the embryo if pregnancy occurs. In this sense, we observed (unpublished data) that in six out of ten women in coma (aged between 28 and 39 years, Glasgow coma scale score 7–11, time in coma 2–10 years), produced by a traumatic brain injury or stroke, menstrual cycles reappeared and normalized (the patients were amenorrheic since their injury occurred), 10–18 months after beginning the administration of the hormone and rehabilitation in our Medical Center. This effect must be attributed to GH, since it is unlikely that the rehabilitation followed may have effects at the ovarian level or on the hypothalamic-gonadotropic axis, and there are no data indicating that this could be the case.

### Growth Hormone and Menopause

The pituitary secretion of GH suffers strong changes throughout human life: very high release during the first year of life, a decrease during childhood followed by a new high release when puberty begins and then a progressive decrease in the amount of hormone released in each secretory pulse [for review, see (2)]. After age 20, more or less, GH secretion is progressively declining by one-half every 7–12 years (73), and it is accompanied by a decline in plasma levels of IGF-1, although its decrease is lesser than that occurring with GH (74). Therefore, at age 50, more or less, GH secretion is residual, if it exists, and it leads to significant changes in body composition, such as reduced muscle mass, increased adiposity, reduced energy, decline in sexual activity, and increased cardiovascular risk, among other symptoms (75). Given the beneficial effects of GH, the question is: why does GH secretion declines alongside aging? Deconvolution analysis of data obtained from blood sampling every 20–30 min in humans indicate that there are age-related alterations in the hypothalamic control of GH secretion, its modulation by gonadal steroids, and in GH autofeedback, leading to significantly decreased GH secretion in elder people (76, 77). This supports the pioneering study conducted in 1985 in which it was demonstrated that the integrated concentration of GH during 24 h was strongly affected by the age of the individual (78) and also reinforces the role played by gonadal sex steroids in the neuroregulation of GH (Figure 4A).

Although GH secretion begins to decrease early in life, menstrual cycles are normally maintained until later ages (45–50 years). Menopause, the final interruption of menstrual cycling, occurs when the pool of ovarian follicles is fully depleted. This leads to a high increase in the production of Gns, with plasma FSH values that double or triple those of LH (Figure 4A). This reflects the loss of ovarian inhibit production, the main negative regulator of FSH secretion, and also the deficient production of estrogen by the menopausal ovaries, which leads to a greater release of LH. Consequently, although throughout this review, we provide a number of data showing the positive effects of GH in the ovaries and its role as co-gonadotropin, it seems that age-related GH deficiency is not the reason for the interruption of the normal ovarian function. In fact, the menopausal ovaries still release a small amount of E2, and the administration of 1 mg of this estrogen twice daily during 7–10 days to healthy post-menopausal women induces a two-fold amplified mass of GH secreted per burst, and an augmented amplitude and mesor of the 24-h rhythm in GH release (79). The effects of E2 on GH secretion after menopause were confirmed in a double-blind, controlled study in which plasma GH was analyzed before and after somatostatin infusion in healthy post-menopausal women treated with placebo or with an inhibitor of aromatase or a selective inhibitor of the E2 receptor. In this study a 1-h GH peak rebound after somatostatin infusion was clearly decreased during both estrogen-deprivation protocols (80). This study concluded that GH secretion after menopause depends on low levels of ovarian estrogens, and also confirms our previous postulate about that the interruption of somatostatin release induces the synthesis and secretion of GH via GHRH (9), and that sex steroids act on GH neuroregulation by inhibiting somatostatin secretion via alpha-2 noradrenergic pathways (9).

A recent study analyzed the effects of the administration of GH over 26 weeks on the nocturnal pulsatile secretion of LH and the levels of sex steroids in a group of healthy post-menopausal women. Their results indicate that GH did not exert any significant effect on the secretory dynamics of LH or plasma levels of LH, nor were plasma levels of sex steroids modified, although IGF-1 did increase significantly (81). This indicates that neither GH nor IGF-1 modulates the hypothalamic-pituitary-gonadal function in older post-menopausal women.

In all, these data indicate that although somatopause and menopause are more or less temporally related, once menopause begins the administration of GH seems to be totally ineffective at the ovarian level, likely related to the depletion of ovarian follicles while aging.

### Growth Hormone and *in vitro* Fertilization in Poor Ovarian Responders

Based in the data presented in this review it is likely that GH administration may be useful as an adjuvant therapy in *in vitro* fertilization (IVF) for poor ovarian responders (POR) unable to get pregnant. In fact, the combined treatment with GH and gonadotropins was already used many years ago with successful results in terms of more follicles developed, more oocytes collected, and higher urinary estrogens in patients with polycystic ovaries (82, 83). Since those studies, several different trials have been conducted combining GH treatment with Gns or human chorionic gonadotropin to induce *in vitro* fertility and embryo transfer in POR, and although some contradictory results have been reported, the overall conclusion is that the addition of GH significantly improves pregnancies in these infertile women and the number of positive results in terms of live birth rate. In this line, a recent study in 62 older women showed that co-treatment with GH led to the preovulatory down-regulation of FSHR, BMPR1B, and increased density of the largest follicles, and improved fertility in these older women in which there was already a significant decrease in the ovarian follicular reserve (84). Another study analyzed the effects of 6-week pretreatment with GH in POR which were submitted to an *in vitro* fertilization treatment. This study, carried out in 380 POR, showed that the administration of the hormone significantly improved the rate of utilization of oocytes and embryo quality increasing the live birth rates, even in older patients who had previously experienced unsuccessful results from classical techniques (85). Moreover, another recent study demonstrated that co-treatment with GH in patients with normal ovarian response significantly increased pregnancy rate (86). Therefore, from these and other studies, it seems to be clear that GH plays a key role in ovarian fertility and Assisted Reproductive Techniques. An extensive and detailed analysis of these effects of GH as adjuvant therapy in IVF and embryo transfer can be seen in the review carried out by Li et al. (87).

## Ovarian Angiogenesis

As is logical, and it has been briefly described before, for a normal ovarian functioning the gland needs to receive an adequate and perfectly regulated blood supply.

Follicles are the main functional structures of the ovaries; they are formed during fetal development and are composed of a single layer of cells, granulosa cells (GCs), that surround the oocyte. These are the primordial, inactive follicles; these GCs are surrounded by another type of cells, the thecal cells (TCs) which will play a very important role, producing sex steroids, in the development of the follicles until their final stage which culminates in ovulation and luteogenesis.

Before the beginning of the reproductive life, a number of primordial follicles have been transformed in primary follicles. These consist in the oocyte surrounded by the pellucid zone and GCs. Around these a basal membrane separates the GCs from the TCs. In each menstrual cycle, a small number of primary follicles grows until, in general, only one of them, the dominant follicle, suffer a proteolytic process that allows the release of the ovule, while the other follicles that had initially begun to evolve together with the dominant follicle, progressively suffer a process of atresia. The question is: why does only one follicle ends the process leading to ovulation in each menstrual cycle?

**Figure 7:**
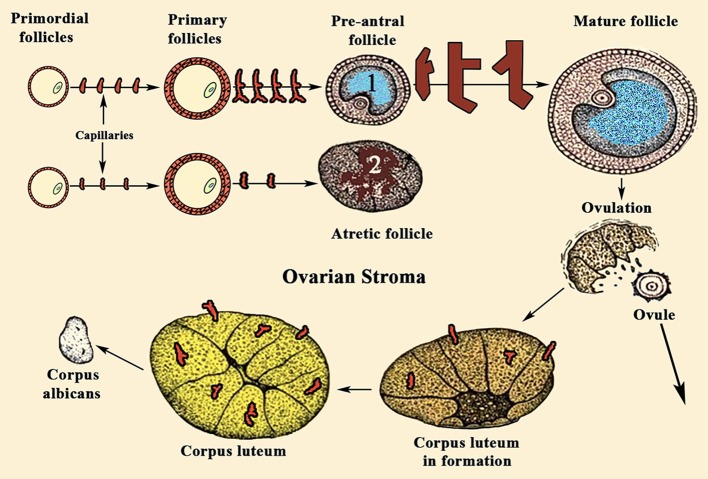
Sequence of ovarian events during a normal menstrual cycle. During a normal menstrual cycle some primordial follicles begin to grow but, usually, only one of them reach the final maturation until it releases the ovule. It depends on the amount of VEGF-A and E2 existing in the follicular fluid. (1) Shows a dominant follicle, in which VEGF-A and E2 levels are quite higher than in the follicles that began to grow with it. These follicles degenerate and become atretic (2). Note the high number of capillaries supplying blood to follicle (1), while the atretic follicle (2) is almost privated of blood supply. VEGF-A, among other factors, is the main responsible for the generation of new small vessels supplying blood to the follicles. Similarly, after ovulation, the formation of the corpus luteum requires an increase in blood supply, as can be seen in the figure.

For years it was believed that this was due to the different aromatase activity that transformed the testosterone produced in the TCs into estradiol (E2) in GCs (Figure 2); the greater the amount of this steroid, the greater the follicular growth capacity, a characteristic of the dominant follicle. Most likely this concept is valid, but: what determines the amount of aromatase in each follicle? Years ago, it was demonstrated that in the adult ovary the vasculature is not distributed uniformly among the ovarian follicles (10). Primordial follicles and slow-growing preantral follicles only have blood supply from vessels existing in the surrounding stroma. This implies that an adequate vascular supply is a rate-limiting step in the selection and maturation of a dominant follicle (88), while those follicles with insufficient blood supply would have limited their growth and would suffer atresia; in fact, the existence of a correlation between increasing levels of the vascular endothelial growth factor A (VEGF-A) and E2 in follicular fluid agrees with the idea that follicles with highest levels of VEGF-A will grow until reaching a preovulatory state (89) (Figure 7). As the follicular development and sex steroids production is under the control of pituitary Gns and some other growth factors, both concepts are compatible. Moreover, this reinforces the important role of angiogenesis in the ovarian function. In fact, blocking VEGF-A effects on the ovary leads to marked decrease of proliferation in the theca of secondary and tertiary follicles and also decreases GCs proliferation and the subsequent production of sex steroids.

### How Ovarian Angiogenesis Is Regulated?

Fibroblast growth factor-2 (FGF-2) was the first factor identified as pro-angiogenic in the ovary (90, 91), being expressed in the mature follicle and corpus luteum. However, given its effects on many different cell types, it is not considered as a factor of interest in the ovarian angiogenesis. In fact, its ovarian expression shows small variations during a menstrual cycle in primates and other mammals. The same occurs with many other pro-angiogenic factors which are not specific of vascular endothelial cells (3).

As stated above, follicular development implies the proliferation and differentiation of GCs; the same occurs with the surrounding TCs. These processes are induced by pituitary Gns, and continue until the dominant follicle, after a sudden and high luteinizing hormone (LH) surge, releases the mature ovule and CL is formed and maturates producing mainly progesterone (P_4_). If there is no conception, this CL undergoes important morphological and functional changes that lead to its regression and formation of a residual structure, as is the corpus albicans (90), in which degeneration of the vasculature exists (88). This succession of events in a menstrual cycle occurs in parallel with rapid and continuous changes in the irrigation and functionality of the ovarian structures involved (92). In fact, in the CL there is a high density of capillaries (Figure 7), as they are microvascular endothelial cells the most abundant cell type in this structure; even more so, each luteal cell is in contact with one capillary (92, 93), most likely to increase the production of P_4_, key for the beginning of pregnancy.

The first factor identified as main responsible of the vascular changes occurring during a menstrual cycle was VEGF-A (94). In the pre-ovulatory follicle, the granulosa layer is avascular, while the theca is strongly vascularized. During follicular development VEGF-A is accumulated, until LH induces proteolytic activity and the basement membrane is degraded; this leads to the release of VEGF-A which induces migration of endothelial cells to GCs, endothelial proliferation and sprouting of pre-existing vasculature, tube formation and recruitment of pericytes, as well as, perhaps in conjunction with other angiogenic factors, vessel stabilization, and maturation (95). These actions of VEGF-A are mediated by the VEGF-A receptor 2 (VEGF-AR2), while VEGF-AR1 seems to play a negative role suppressing signaling through VEGF-AR2.

**Figure 8:**
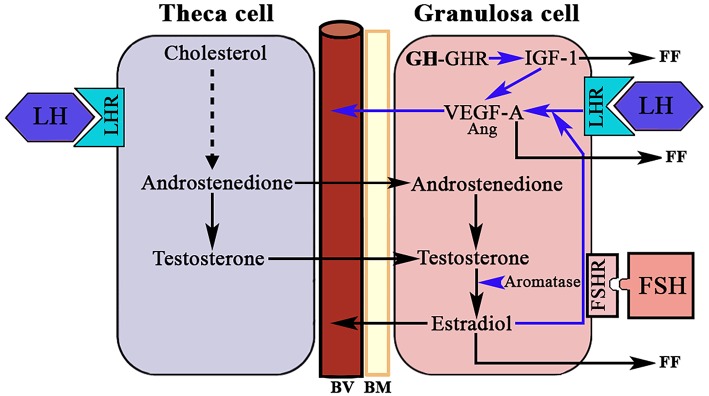
LH, GH/IGF-1, and ovarian angiogenesis. The androgens produced in the theca cell under the stimulation of LH cross to the granulosa cell where, under the stimulation of FSH, they are aromatized to estradiol. In the growing follicle, there are receptors for LH that in combination with the produced E2 induces the expression of VEGF-A (mainly) and angiopoietin 1 (Ang). VEGF-A and Ang 1 induce the formation of new vessels (BV). There is a possibility that the GH produced in the granulosa cells induces the expression of IGF-1 who also favors the production of VEGF-A. VEGF-A and estradiol are released into small blood vessels, and both factors and IGF-1 are also released into the follicular fluid (FF). BV, blood vessels; BM, basement membrane; LHR, LH receptor; FSHR, FSH receptor; GHR, GH receptor; Blue arrows, stimulation.

Another group of endothelial-specific factors detected in the ovary are Angiopoietins (Ang) (96, 97), of which Ang2 seems to act, in the absence of VEGF-A, as an inducer of ovarian vessels destruction. This effect is produced by the binding of Ang2 to the Ang1 receptor Tie2, therefore impeding the binding of Ang1. Thus, Ang2 acts in an opposite manner than the isoform Ang1 who, like VEGF-A, is essential for normal vasculature development (97), and stabilization of newly formed blood vessels. However, in the presence of VEGF-A increased autocrine expression of Ang2 by the vascular endothelium induces angiogenesis. Perhaps these divergences explain the reason by which Ang1 and Ang2 are differentially expressed in the ovary during a normal menstrual cycle. Interestingly, pituitary gonadotropins, in particuar LH, have been demonstrated to be major inducers of angiogenesis and VEGF-A/Ang expression in the ovary. The midcycle surge of LH strongly stimulates VEGF-A and Ang expression in GCs in many species, including primates (98) (Figure 8), while the administration during 3 days of a Gn-releasing hormone (GnRH) antagonist, which leads to the blockade of LH secretion, clearly decreased VEGF-A expression in the CL of primate ovaries (99). However, this effect of LH on ovarian VEGF-A expression may be modulated or be dependent of local ovarian factors, such as ovarian sex steroids (100), as occurs in other tissues. This agrees with the concept that the VEGF-A ovarian expression changes cyclically throughout a menstrual cycle (101).

### Pituitary Gonadotropins and Ovarian Angiogenesis

The effects of LH on the ovarian production of VEGF-A have been confirmed in infertile, but otherwise normal, women to which recombinant LH (rLH) was administered (75 U/day) during the late follicular phase, induced by administration of rFSH, in which the plasma and follicular liquid levels of VEGF-A, and its soluble receptor sFlt-1, were evaluated. The results obtained in this pioneer study indicated that rLH significantly increased the ratio VEGF-A/sFlt-1 in the follicular fluid, indicating that LH induced ovarian follicular angiogenesis (92), while inhibiting VEGF-A by treatment with truncated Flt-1Fc (vascular endothelial growth factor trapA40) leads to the inhibition of angiogenesis during follicular development in primates (102).

Although the effects of LH on ovarian angiogenesis are clear, some data indicate that FSH may also play a role in this process. For instance, it has been demonstrated that the addition of 0.3 IU/mL of FSH during ovarian cryopreservation by vitrification increased the revascularization and follicular survival for mouse ovarian grafts up-regulating angiogenesis and ovarian survival factors (103); this may preserve ovarian fertility avoiding the follicular damage produced by chemotherapy and/or radiotherapy. Recently, it has been demonstrated that interleukin-6 (IL-6), a major factor in the regulation of VEGF-A expression (104), also promotes the expression of VEGF-A induced by FSH in bovine GCs (105), suggesting that there is a synergistic relationship between FSH and IL-6 in the regulation of VEGF-A expression. This effect of IL-6 on VEGF-A is dependent on the promotion by IL-6 on the VEGF-A regulators HIF-1α (hypoxia inducible factor-1α) and COX2 (cyclooxygenase2), since their inhibition significantly decreases VEGF-A expression in GCs. In any case, it seems that a direct role of FSH in ovarian angiogenesis is restricted to particular non-physiological situations.

### Growth Hormone and Ovarian Angiogenesis

Among the factors regulating VEGF family expression in humans GH plays a pivotal role, either directly or by inducing the expression of other proangiogenic factors, such as the Insulin-like growth factors (IGF-1 and IGF-2), FGF-2, epidermal growth factor (EGF), among others; moreover, GH is able to interact with receptors for Prolactin (PRL), which also is able to induce proangiogenic effects [for review, see (106)]. At this point, it is important to note that the pituitary secretion of GH is strongly potentiated by sex steroids, mainly E2, which in its free form (fE2) directly reaches the central nervous system (CNS), or is formed at this level from the hypothalamic aromatization of testosterone (9).

The possibility exists that systemic GH could induce ovarian VEGF-A expression, but this has not been demonstrated; perhaps because the own GH and its receptor are produced in the ovary in humans and bovines (11, 13, 107). Moreover, the production of GH by ovaries is higher in GCs and oocytes, avascular follicular compartments, separated from the systemic circulation by the basal lamina (8) (Figure 4). Local expressions of GH and GHR have also been detected in the chicken ovary during sexual maturation in hens (108, 109) and fishes (110). Ovarian GH would act in an autocrine/paracrine way, so the hormone could play a role in the regulation of ovarian angiogenesis, as suggested by the fact that ovarian expression of the GH gene increases during follicular development (11), but significantly decreases when immature follicles reinitiated meiosis (111). The cyclic expression of follicular GH shows parallelism with the expression of follicular GHR in some species analyzed, including humans (13, 108, 112). If this temporary pattern of ovarian GH-GHR correlates with changes in ovarian VEGF-A during a normal menstrual cycle, has not yet been established. However, the fact that the expression of VEGF-A in the follicle is related to the size of the follicle itself (Figure 7) is crucial to understand why angiogenesis is important and why GH, the main hormone involved in tissue growth, should play an important role in the production of VEGF-A in the ovary (89).

How the ovarian synthesis of GH is regulated is not known, although, as described above, GH-releasing hormone (GHRH) mRNA and GHRH receptor were found many years ago in humans and rats ovaries (112, 113). However, differently to that occurring at the hypothalamic-pituitary axis, ovarian GHRH does not seem to stimulate the synthesis and secretion of ovarian GH. In fact, recently it has been reported that a GHRH homodimer up-regulates the ovarian GHRH receptor increasing the development and maturation of follicles without affecting ovarian GH production, at least in an animal model (114). Another important GH secretagogue, such as ghrelin, has been found to increase GH secretion, but not GH synthesis, in porcine follicles *in vitro* (115), and ghrelin and its receptor have been found in the ovaries of pig and hen (116, 117). However, administration of ghrelin does not induce ovarian GH release in cultured ovaries in chicken (118). Paradoxically, ovarian GH stimulates ghrelin synthesis and secretion (115).

It cannot be ruled out that GH can act on the ovarian angiogenesis through one of the multiple mediators of the actions of the hormone, mainly IGF-1 (119). There is an ovarian production of IGF-1 and its receptor that induces angiogenesis by stimulating the production of VEGF-A in luteal cells, and also by stimulating the proliferation and differentiation of endothelial cells (120, 121). IGF-1 can act directly or as a mediator of the proangiogenic effects of GH (119). GH stimulates IGF-1 expression in rat and porcine granulosa cells (61, 122), and IGF-1 antibodies block the effect of GH on oocytes maturation in rat follicles (123). However, a study indicated that GH administration does not induce the synthesis of IGF-1 in human pre-menopausal ovaries (124), although previous studies showed that GH increases IGF-1 in follicular fluid in a number of species, including humans (Figure 5) [for review, see (14)]. Therefore, the GH effects on ovarian IGF-1 production may be exerted via endocrine or auto/paracrine actions.

Other proangiogenic growth factors, present in the ovary and whose transcription is induced by GH, such as FGF-2 and EGF, among others, seem not to play any key role on ovarian angiogenesis, although they can contribute to it; rather they participate in steroidogenesis, follicular development, and luteal function (125).

**Figure 9:**
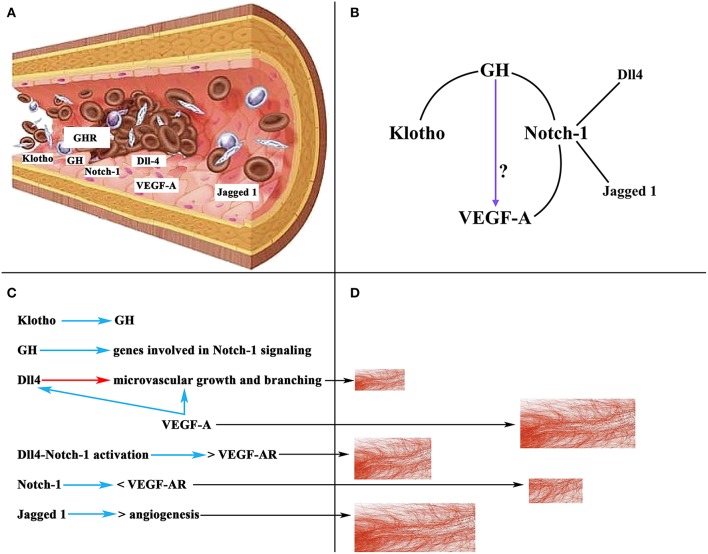
Schematic representation of the action of VEGF-A, Notch-1 and its ligands on ovarian angiogenesis. **(A)** Section of an ovarian vascular vessel showing the expression of some factors involved in ovarian angiogenesis: Klotho, VEGF-A, Notch-1 and its ligands Dll4 and Jagged 1, the receptor for GH (GHR), and presumably GH. **(B)** Relationships between Klotho, GH, Notch-1, and VEGF-A. These lead to the possibility that GH can act directly on the induction of the expression of VEGF-A in the endothelium of ovarian microvessels. **(C)** Summary of the actions of the factors mentioned in **(A)**. Blue arrows, stimulation; Red arrow, inhibition; <, decrease; >, increase; VEGF-AR, receptor for VEGF. **(D)** Schematic representation of the growth of ovarian microvessels produced by the action of each one of the factors showed in **(C)** (black arrows).

Of interest here is the relationship between GH, Notch-1, and VEGF-A. GH has been shown to regulate the ovarian expression of some genes involved in Notch-1 signaling to induce repair and regeneration of ovaries in mice with premature ovarian failure, as well as to induce E2 secretion and oocyte maturation (126). We will not analyze here the important effects that Notch-1 plays in the organism, but only the most important ones on angiogenesis in the ovary (127). In mice, during the follicular phase Notch-1 is expressed in the endothelium of the thecal layer, while in the luteal phase Notch-1 has been detected in endothelial cells from new vessels of the CL and mature vessels of the thecal layer. In primates, two important Notch-1 ligands, such as delta-like protein Dll4 and Jagged 1 (126, 128), regulate angiogenesis directly in the endothelium, being specifically expressed at sites where this angiogenesis occurs (129). Dll4 regulates microvascular growth and branching induced by VEGF-A to prevent excessive branching that could lead to vascular dysfunction (130), whereas the blockade of Dll4 with an anti-Dll4 monoclonal antibody leads to an increase in luteal angiogenesis and a greater density of microvessels in the primate ovary (131). In turn, inhibiting Jagged 1 induces anti-angiogenic effects (132). Based on these data it is evident that Notch-1 plays an important role in ovarian angiogenesis. Curiously, there is a strong relationship between VEGF-A and Notch signaling. VEGF-A increases Dll4 expression in endothelial cells *in vitro* (133), and Dll4-Notch-1 activation induces an increase in the expression of VEGF-A receptors in cultured endothelial cells (134), although activation of Notch-1 alone induces a reduction in the expression of VEGF-A receptor 2 (134). All this may be related to the need to form and maintain an adequate number and functionality of the ovarian blood vessels for a normal ovarian function in each of the phases of a menstrual cycle. In fact, ovarian angiogenesis is abnormal in women with polycystic ovarian syndrome; in these patients, there is an increase in follicular vascularization and vascular permeability (135). The excessive ovarian vascularization is responsible for the pathologic characteristics of the syndrome (127). Figure 9 shows a schematic representation of the effects of VEGF-A, Notch-1 and its ligands on ovarian angiogenesis.

As stated above, there is no evidence to show that GH induces the ovarian expression of VEGF-A; however, the possibility exists that ovarian vessels produce GH and this hormone promotes the expression of VEGF-A. In fact, the vascular endothelium is an important gland of internal secretion that produces a series of growth factors, including GH (136) (Figure 9A), that act in a paracrine/autocrine fashion. In addition, there is a specific GHR in the vascular endothelium (137, 138) (Figure 9A), and the damaged vascular endothelium produces Klotho, which stimulates the pituitary secretion of GH, but perhaps also the production of endothelial GH to repair the vascular endothelium when it is damaged (3). Therefore, given the explained relationships between Notch-1 and VEGF-A, and Notch-1 and GH, and Klotho and GH, it seems feasible that GH may directly participate in the regulation of ovarian angiogenesis (Figure 9B).

In summary, it is clear that adequate angiogenesis is critical in each of the phases that a primary follicle has to follow until it is transformed in an ovulatory follicle, ovulation occurs, and CL is formed and maintained until pregnancy takes place. Abnormal angiogenesis not only impedes the physiological evolution of this process but also may be the cause of pathologies such as infertility, polycystic ovarian syndrome and even ovarian cancer. Ovarian angiogenesis is very complex, and although VEGF-A plays a key role in it there are many factors, still little known, that can influence the actions of this protein both positively and negatively.

## Conclusions

In conclusion, it is clear that GH, expressed in the ovary and/or systemic GH, is very important in all stages of ovarian development and normal functioning until menopause. The ovarian effects of GH can be exerted by the hormone itself and/or through its mediators. Of interest is the bidirectional inverse relationship between the actions of GH and BMP at the ovarian level, as well as their actions on the secretion of gonadotropins. It is likely that GH can be a very important factor as adjuvant therapy for IVF and embryo transfer in infertile women poor ovarian responders. In addition, ovarian angiogenesis plays a key role in the normal ovarian functioning since puberty begins until menopause. The formation and functionality of the ovarian vessels depend mainly on VEGF-A, although Angiopoietins also play a role in ovarian angiogenesis. Ovarian expression of VEGF-A is regulated by LH together with sex steroids, but GH also appears to be (directly or indirectly) actively involved in this process.

## Author Contributions

JD conceived, structured, and wrote the text. DC contributed to writing the section devoted to Ovarian Angiogenesis.

### Conflict of Interest Statement

The authors declare that the research was conducted in the absence of any commercial or financial relationships that could be construed as a potential conflict of interest.
